# Pile-up transmission and reflection of topological defects at grain boundaries in colloidal crystals

**DOI:** 10.1038/s41467-020-16870-w

**Published:** 2020-06-17

**Authors:** Xin Cao, Emanuele Panizon, Andrea Vanossi, Nicola Manini, Erio Tosatti, Clemens Bechinger

**Affiliations:** 1 Fachbereich Physik, Universität Konstanz, 78464 Konstanz, Germany; 2 International School for Advanced Studies (SISSA), Via Bonomea 265, 34136 Trieste, Italy; 3 CNR-IOM Democritos National Simulation Center, Via Bonomea 265, 34136 Trieste, Italy; 4 Dipartimento di Fisica, Università degli Studi di Milano, Via Celoria 16, 20133 Milan, Italy; 5 The Abdus Salam International Centre for Theoretical Physics (ICTP), Trieste, Italy

**Keywords:** Colloids, Structure of solids and liquids

## Abstract

Crystalline solids typically contain large amounts of defects such as dislocations and interstitials. How they travel across grain boundaries (GBs) under external stress is crucial to understand the mechanical properties of polycrystalline materials. Here, we experimentally and theoretically investigate with single-particle resolution how the atomic structure of GBs affects the dynamics of interstitial defects driven across monolayer colloidal polycrystals. Owing to the complex inherent GB structure, we observe a rich dynamical behavior of defects near GBs. Below a critical driving force defects cannot cross GBs, resulting in their accumulation near these locations. Under certain conditions, defects are reflected at GBs, leading to their enrichment at specific regions within polycrystals. The channeling of defects within samples of specifically-designed GB structures opens up the possibility to design novel materials that are able to confine the spread of damage to certain regions.

## Abstract (web summary)

The plastic flow of crystals takes place via the elementary flow of topological defects and is strongly influenced by the presence of grain boundaries. Here, the authors show how the atomic structure of grain boundaries affects the dynamics of interstitial defects driven across monolayer colloidal polycrystals.

## Introduction

The plastic deformation of crystalline materials typically takes place via the elementary flow of topological defects such as dislocations and interstitials^1–5^. Therefore, the dynamics of such defects under external stress is of central importance for understanding the mechanical behavior of crystals. In contrast to their rapid propagation within single crystals, the motion of the defects is severely influenced by grain boundaries (GBs) in polycrystals, leading to a mechanical reinforcement of polycrystalline materials which increases with the inverse average grain size^6–10^. This empirically observed Hall–Petch relation has been explained with the GB-assisted accumulation of defects, which leads to an increasing yield strength^9,10^. Evidence for this pile-up mechanism is provided by electron-microscopy experiments, where defects, which have been created by indentation of nanometer-sized tips, are observed to accumulate at GBs^11–14^. While the interactions of defects with GBs have been intensively studied in atomic simulations^15–19^, such detailed and particle-resolved investigations of the GB-defect interactions are limited from the experimental side. In particular, how the inhomogeneous atomic GB structure locally influences the incoming defects has not been thoroughly investigated in experiments. Such knowledge, however, is mandatory to provide quantitative relationships between the structure and the mechanical properties of polycrystalline materials.

Here we report an experimental and theoretical study to unravel the properties of driven interstitial defects in polycrystalline colloid monolayers with single-particle resolution. Experimentally, this is achieved by injecting colloidal particles into a colloid monolayer interacting with a patterned triangular substrate emulating polycrystalline grains with various GB topology. Colloids are “magnified atoms” with a length scale ~4 orders of magnitude larger than atoms and time scale ~6 orders of magnitude slower^20^. Due to the easily accessible time and length scales and the possibility of tuning the relevant microscopic interactions in colloid experiments, colloidal systems have been established as versatile analogic models to provide detailed insight at single-particle level, e.g., in phase transitions, nanofriction, clogging and jamming in the flow of particles hindered by obstacles^21–24^. When injecting interstitial defects into the system by an external driving force, we observe that their motion is strongly hampered only at specific positions of the GB, leading to their distortion and splitting upon crossing the GB. Below a critical driving force the defects are not able to cross GBs, which leads to their accumulation (pile-up) at these locations. A Hall–Petch-like relation is recovered by measuring the critical force as a function of the grain size. Even though our investigations pertain to two-dimensional colloid monolayers where the detailed structure and dynamics of the defects are very different, the observed Hall–Petch strengthening relation and position-depend dynamic behaviors are qualitatively similar to those observed in defect-GB interactions in three-dimensional real materials^15–19^. Remarkably, we also find that, under certain conditions, the GB can reflect incoming defects. Their confinement to specific regions of the polycrystalline sample suggests the fabrication of polycrystalline monolayers with direction-dependent mechanical properties.

## Results

### Experiment

**Fig. 1:**
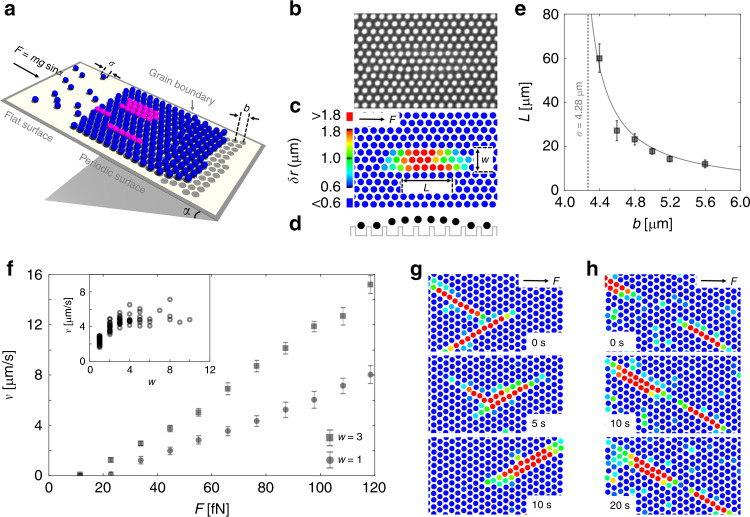
Realization of interstitials in colloidal experiments. **a** Illustration of the experimental setup. Interstitials are formed by injecting colloidal particles into a crystalline phase on top of a patterned surface with periodic domains separated by well-defined GBs. **b** The interstitials observed in colloidal crystals. Its crystallographic nature is shown in detail in Supplementary Fig. 3. **c** Same as (**b**) but with particles color coded by their distances *δr* to the nearest potential minimum. **d** Sketch of the one-dimensional particle distribution of an interstitial over the substrate pattern. **e** The length *L* of the interstitial defects (defined as those particles with *δr* > 1.2 µm) as a function of the substrate periodicity *b*. The dashed line indicates the size *σ* of the spheres. The solid line is a fit to the equation *L*/*σ* = σ*/(*b* − σ*), with fitted σ* = 4.09 ± 0.02 µm < *σ* = 4.28 µm. **f** The interstitial velocity *v* as a function of *F* for lateral size *w* = 1 and *w* = 3. Depinning thresholds of about 20 and 10 fN are observed, respectively. As expected of a Peierls–Nabarro barrier^26^, this threshold value is much smaller than the threshold to move isolated particles across the same pattern, see Supplementary Fig. 4 (Inset). The interstitial velocity as a function of *w*, with fixed *F* = 45 fN. **g** Two *w* = 1 interstitials collide and merge into a *w* = 2 interstitial; *F* = 55 fN. **h** A moving *w* = 2 interstitial approaches an immobile *w* = 1 interstitial from behind. When they come close, the *w* = 2 interstitial pushes the *w* = 1 interstitial forward; *F* = 23 fN. Color code in (**g**) and (**h**) is same as in (**c**). All error bars are standard deviations of the corresponding data points.

Our experimental setup is illustrated in Fig. 1a. Silica particles with hard-sphere interaction and diameter *σ* = 4.28 μm are driven by gravity *F* = *mg* sinα across a flat surface (reservoir) toward a patterned surface with hexagonal symmetry and lattice spacing *b* > *σ*. Specifically, this periodic substrate is patterned with cylindrical wells created by photolithography. Each well (with radius *r* ≈ 1.6 μm and depth *h* ≈ 0.5 μm, see Supplementary Fig. 1) can be occupied by one particle only. When reaching the patterned area, the colloids become trapped in the wells, eventually forming a hexagonal crystal with lattice spacing *b*. Under the gravitational driving force, extra “interstitial” colloidal particles can be injected from the reservoir into the crystal phase, which leads to the formation of zero-dimensional (point like) and quasi-one-dimensional (i.e., aggregated) interstitials (see Supplementary Movie 1). These interstitials propagate along the lattice vector being closest to the driving force. To study the interaction of interstitials with GBs, we construct patterns of substrate wells arranged as single-crystalline domains separated by well-defined GBs (Supplementary Fig. 1). More experimental details are in the “Methods” section.

### Interstitial defects in uniform lattice

Before discussing the behavior of interstitials near GBs, we summarize their properties on a uniform, i.e., single domain, lattice. Figure 1b shows an optical image of an interstitial defect, which is formed by three interstitial particles. To better visualize the corresponding strain field, we color code each particle by its distance *δr* to the nearest substrate well in Fig. 1c, see “Methods” for the calculation of *δr*. It reveals a crowdion (a specific type of interstitial) configuration where the strain caused by the interstitial is confined to an elongated area with length *L* ≈ 6.0*b* and *w* = 3 lines of particles wide. Given the mismatch between the particle size *σ* and the substrate lattice spacing *b*, the length *L* of the interstitials varies as shown in Fig. 1e. In the rest of the paper, we select *b* = 4.6 μm, generating interstitials of relative length *L/b* ≈ 6.0, which matches the size of crowdions in metallic crystals^25^. As sketched in Fig. 1d, the features and motion of our interstitials resemble the topological solitons described by the Frenkel–Kontorova model^26,27^. In addition to the Peierls–Nabarro (PN) potential arising due to the interaction with the underlying periodic surface, our interstitials experience a lateral friction force when moving through the lattice due to interactions with particles in neighboring motionless lines (Supplementary Fig. 2). Since the lateral friction force exists only at the boundary between the interstitial and the undistorted lattice, the interstitials become faster with increasing width, as shown in Fig. 1f, which compares the force-dependent velocity of interstitials with *w* = 1 and 3. Further evidence that the lateral friction of interstitials is essentially given by their boundary with the surrounding is also provided by the fact that the interstitial velocity rapidly saturates with increasing *w* (inset Fig. 1f). An immediate consequence of this lateral friction is their aggregation once they approach each other from different directions. This is exemplarily shown in Fig. 1g and Supplementary Movie 2 for the case of two interstitials with *w* = 1 merging into a single one with *w* = 2. Such aggregations lead to reduced collisions between interstitials and therefore enhance the average interstitial velocity (Supplementary Movie 3). In contrast, repulsion is observed (Fig. 1h, Supplementary Movie 4) when a fast (*w* = 2) interstitial approaches a slower one (*w* = 1) along the same line. This situation is similar to the interaction of running kinks in one-dimensional systems, which also exhibit a repulsive interaction due to the overlap of the compressive strain fields^28^.

### Driving interstitial defects across small-angle GBs

**Fig. 2:**
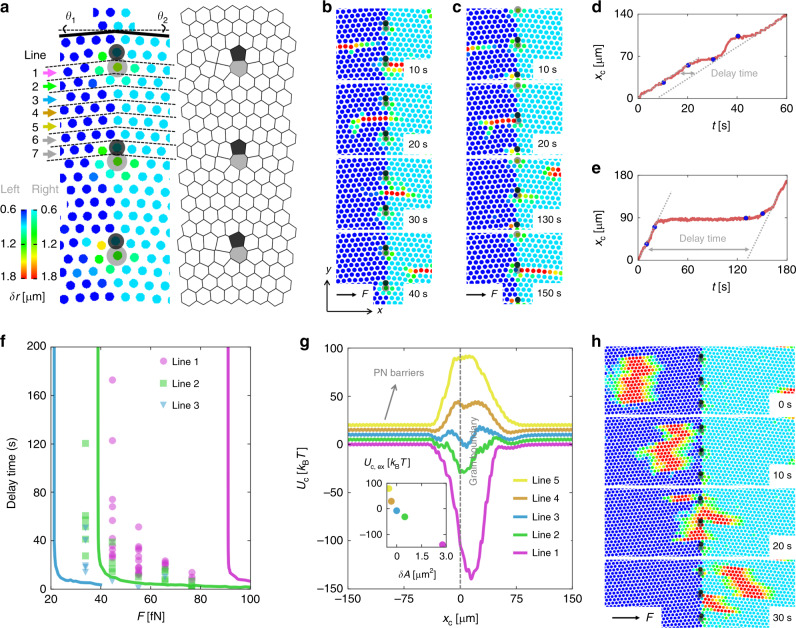
Slowing down of interstitials by small-angle GBs. **a** Left: color-coded experimental images showing particle positions near a GB (*θ*_1_ = *θ*_2_ = 4.72°). To highlight the position of the GB, slightly different color maps are used at the two sides of the GB. Arrows indicate the seven different lattice lines. Right: Voronoi tessellation of the left image, pentagons are filled with black, heptagons are filled with gray, hexagons are not filled. **b**, **c** Snapshots showing a *w* = 1 interstitial going across the GB via line 4 and line 1, respectively at *F* = 45 fN. The particle color code and the highlight of 5–7 pairs are as in (**a**). **d**, **e** The center-of-mass displacements *x*_c_ of the interstitials in (**b**) and (**c**), respectively, as a function of time *t*. The dots correspond to the snapshots in (**b**) and (**c**), respectively. The delay time is defined as the horizontal distance between the trajectories before and after the delay at GB. **f** The measured delay time as a function of *F* for *w* = 1 interstitials following different lattice lines across the GB. For line 1, the delay time diverges around *F* = 45 fN. For the other lines avoiding the 5–7 pairs, the delay time, as well as the critical force, is smaller. Lines are results from corresponding (same color) *T* = 0 simulations of the experimental data points. **g** The potential energy *U*_c_ of an interstitial with *w* = 1 versus its center-of-mass position along lines 1–5. The GB is at *x* = 0. The curves are shifted from one another by 5 *k*_B_*T* for clearance. Due to the lattice distortion at the GB, the potential energy deviates significantly from the regular-lattice PN potential, over an interval from *x* ~ −20 µm to *x* ~ 40 µm, consistent with the total length of the interstitial. (Inset) The extreme energy *U*_c,ex_ at the valley or peak as a function of relative Voronoi area *δA* = *A*_GB_ − *A*_0_ of lattice points at the GB. **h** At *F* = 45 fN, a *w* = 14 interstitial is being spliced when traveling through this GB. The particle color code and the highlight of 5–7 pairs are as in (**a**).

The dynamics of interstitials becomes strongly affected by the presence of a GB. In general, GBs in a two-dimensional crystal are characterized by two angles *θ*_1_ and *θ*_2_ indicating the lattice orientations on each side of the boundary (Fig. 2a). To create equilibrium low-energy GBs, however, one also has to consider the structural relaxation of the two domains when bringing them into contact. It has been shown that equilibrium GB configurations in two dimensions with lowest energy can be constructed using a method based on centroidal Voronoi tessellation^29^. Supplementary Fig. 1 shows some examples of a substrate containing GBs with different values of *θ*_1_ and *θ*_2_, which have been realized according to the centroidal Voronoi-tessellation method. Notably, the GBs consist of an almost linear sequence of localized defects, which are characterized by pairs of fivefold and sevenfold coordinated potential wells (5–7 pairs). Between such pairs, the lattices remain almost undistorted.

When a colloidal monolayer is absorbed on such a structure, it closely matches the underlying substrate geometry. This can be seen from the small particle displacements *δr* which are exemplarily shown in Fig. 2a for a symmetric GB with *θ*_1_ = *θ*_2_ = 4.72°. The Voronoi tessellation of the particle positions clearly indicates the positions of the 5–7 pairs of the underlying substrate. When interstitials are inserted and driven perpendicular to the GB, their behavior strongly depends on whether they hit a 5–7 pair or cross the GB in between. When an interstitial passes the only marginally distorted GB region between 5–7 pairs, its velocity remains almost unaffected (Fig. 2b, d and Supplementary Movie 5). Opposed to that, a pronounced time delay of about 130 s in the interstitial trajectory is found when crossing the GB at a 5–7 pair (Fig. 2c, e and Supplementary Movie 5). In Fig. 2f, we show the measured time delay as a function of the driving force for three different crossing points at the GB as indicated in Fig. 2a. With increasing distance from a 5–7 pair, the measured (symbols) delay times systematically decrease in qualitative agreement with numerical simulations (lines, see “Methods”). The deviations from the experiments are possibly due to out-of-plane particle motions and the colloid’s polydispersity which are not considered in the simulations. To understand why the delay time strongly depends on the position where the particle passes through the GB, we have numerically calculated the potential energy *U*_c_ of an interstitial as a function of its center-of-mass distance *x*_c_ to the GB. As seen in Fig. 2g, *U*_c_ is strongly non-monotonic at the GB. In particular when the interstitial passes near a 5–7 pair, the spatial variation of *U*_c_ becomes most pronounced, which explains why the delay time is largest for these regions. The inset of Fig. 2g illustrates the correlation between the depth/height of *U*_c,ex_ and the Voronoi-area deviation *δA* = *A*_GB_ − *A*_0_, where *A*_GB_ is the Voronoi area of the GB lattice point, and *A*_0_ is the Voronoi area of a regular lattice point. When *δA*_g_ < 0, i.e. the lattice is locally compressed, *U*_c,ex_ is positive and the GB acts as a barrier. For *δA*_g_ > 0, i.e. when the lattice is locally expanded, *U*_c,ex_ is negative and the GB acts as a trap. Far away to the left and right of the GB, *U*_c_ displays a tiny oscillation whose wavelength is identical to the periodicity of the substrate. The amplitude of this oscillation corresponds to the PN potential for an interstitial moving across a perfect periodic lattice. As a result of the strong variation of the potential barriers along the GB, interstitials with widths comparable with or even larger than the spacing between 5–7 pairs display a rather complex behavior crossing a GB. This is exemplarily shown in Fig. 2h and Supplementary Movie 6, displaying the dynamics of an interstitial with *w* = 14. Upon approaching the GB, its front becomes distorted and eventually splitted when passing the GB.

### Pile-up of interstitial defects at large-angle GBs

**Fig. 3:**
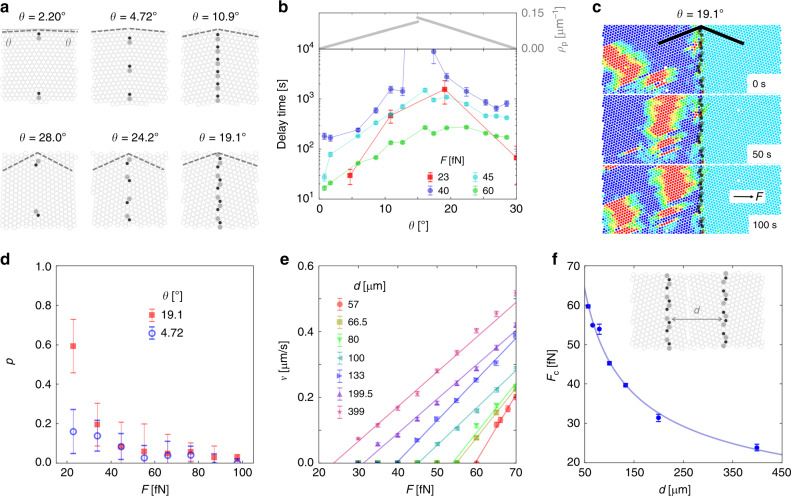
Interstitial pile-up at GBs with large misalignment angles. **a** The structure of GBs at various values of *θ* = *θ*_1_ = *θ*_2_. The 5–7 pairs are highlighted as shaded regions. **b** (bottom) The average delay time as a function of *θ* in experiments (squares) and simulations (circles) at different driving force *F*. (top) The density of 5–7 pairs, Eq. (1). **c** The pile-up of broad interstitials at the *θ* = 19.1° GB in experiment. *F* = 23 fN. The particle color code and the highlight of 5–7 pairs at the GB are as in Fig. 2a. **d** The pile-up ratio *p* as a function of driving force *F* at *θ* = 19.1° and *θ* = 4.72°. **e** The average interstitial velocity *v* as a function of the driving force *F* at various grain sizes *d* in simulation. All GBs are symmetric with *θ* = 19.1° as illustrated in (**f**) inset. Lines are linear fittings *v* = *μ*(*F* – *F*_c_) of the corresponding data points. **f** The critical force *F*_c_ as a function of grain size *d*. Solid line represents *F*_c _= *F*_0_ + *k*/*d*^1/2^ with fitted *F*_0_ = 1 ± 2 fN and *k* = 450 ± 20 μm^0.5^. (inset) Illustration of a polycrystal with grain size *d*. All error bars are standard deviations of the corresponding data points.

On average, the total slowing down of interstitials at GBs strongly depends on the density *ρ*_p_ of 5–7 pairs along the GB, which is a function of the GB misalignment angles, as illustrated in Fig. 3a. For symmetric GBs *θ* = *θ*_1_ = *θ*_2_, *ρ*_p_ is:

1 $$\begin{array}{l}{\mathrm{ }}\\ \begin{array}{*{20}{c}} {\hskip-48pt}{\rho _{\mathrm{p}}\left( \theta \right) = 2\,{\mathrm{sin}}\,\theta /{b},} & {\hskip 4pt}{0^\circ {\,} < {\,} \theta {\,} < {\,} 15^\circ } \\ {\rho _{\mathrm{p}}\left( \theta \right){\mathrm{ }} = {\mathrm{ }}4\,{\mathrm{cos}}\left( {{\it{\uptheta }} + 60^\circ } \right)/\surd3b,} & {15^\circ {\,} < {\,} \theta {\,} < {\,} 30^\circ } .\end{array}\end{array}$$

Eq. (1) is plotted in Fig. 3b (top). The discontinuity at *θ* = 15° stems from the geometrical origin of the defects, and is discussed in the Methods section, alongside the derivation and extension of Eq. (1) to GBs with arbitrary orientations. Near *θ* = 0° and 30° the two grains are almost perfectly aligned, resulting in dilute 5–7 pairs at the GB. The density of 5–7 pairs becomes largest for *θ* = 15°, where the misalignment of the two grains is maximum. Figure 3b (bottom) reports the average delay time (“Methods”) of interstitials when they traverse a symmetric GB of angle *θ*. The results show that the delay time reaches a maximum near *θ* = 15°. Therefore, the denser the 5–7 pairs, the longer the delay time is. With *θ* = 19.1°, the density of 5–7 pairs is so large that a pile-up of interstitials is observed, as shown in Fig. 3c and Supplementary Movie 7, by gradually lowering the driving force toward *F* = 23 fN. To quantitatively describe this pile-up, we define a pile-up ratio *p* (“Methods”) that describes the strength of pile-up. Figure 3d reports the observed *p* as a function of *F* for the *θ* = 19.1° GB and for the *θ* = 4.72° GB. For the *θ* = 19.1° GB, *p* reaches a relatively high value when *F* < 30 fN. Instead, for the *θ* = 4.72° GB, *p* remains small across the entire range of experimental driving forces: generally we observe little or no pile-up against small-angle GBs.

Together with the accumulation of interstitials near GBs, mechanical stress gradients are expected to increase in these regions. Accordingly, the spatial distribution of internal stress should become more homogeneous with decreasing grain size and thus leads to an increased yield stress^9,10^. To verify this, in Fig. 3e we show *v* as a function of *F* for different grain sizes *d* in numerical simulations for the *θ* = 19.1° GB, while keeping the total size of the system and the density of interstitials fixed. *d* is the spacing between two successive GBs as shown in the inset of Fig. 3f. The smaller the value of *d*, the more numerous GBs the interstitials has to cross. Figure 3f shows the critical force *F*_c_ as a function of grain size *d*. Under such force, all interstitials are effectively stuck (pile-up) at one of the GBs. Interestingly, the data in Fig. 3f can be well fitted (solid line) to the Hall–Petch relation *F*_c_ = *F*_0_ + *k*/*d*^1/2^, which describes the strengthening of materials at small grain sizes due to the increasing yield stress with decreasing separations between GBs.

### Reflection of interstitial defects by GBs

**Fig. 4:**
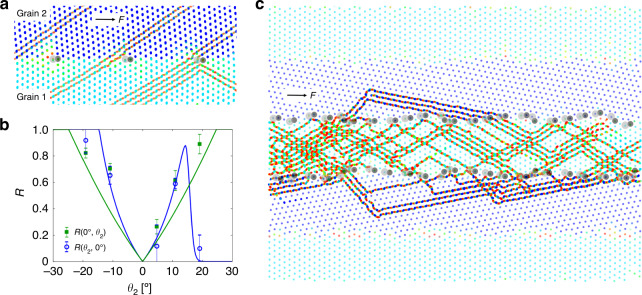
Reflection of interstitial by nonsymmetric GBs. **a** Superposition of particle positions near a GB (*θ*_1_ = 0°, *θ*_2_ = 4.72°) over 80 s time periods, trajectories of three interstitials (*w* = 2, 2, 4 respectively) are clearly revealed. The particles are color coded like in Fig. 2. The 5–7 pairs at the GB are highlighted. *F* = 66 fN. **b** The reflection rates as a function of *θ*_2_ for *R*(0°, *θ*_2_) and *R*(*θ*_2_, 0°), *F* = 66 fN. Points are experimental data. Lines represent Eq. (2) with parameters *b* = 4.6 µm, *k*_B_*T* = 4.14 zJ. *R*(0°, *θ*_2_) is much larger than *R*(*θ*_2_, 0°) when *θ*_2_ > 15°, this makes it possible to confine interstitials within grain 1. Error bars are standard deviations of the data points. **c** The superposition of particle positions in a striped pattern in experiments over a 1000 s time period. The 5–7 pairs at the GB of the inner stripe are highlighted. *F* = 66 fN. The four GBs (from bottom to top) have GB angles (0°, −19.1°), (19.1°, 0°), (0°, 19.1°) and (−19.1°, 0°), respectively.

In addition to the observed slowing down and pile-up of interstitials when crossing GBs, reflection of interstitials can also occur. This is shown in Fig. 4a where part of an interstitial with *w* = 4 is being reflected after hitting a 5–7 pair at the GB. The reflection probability *R*(*θ*_1_, *θ*_2_) for interstitials in the *θ*_1_ side approaching the *θ*_2_ side (and likewise the transmission probability 1 − *R*) depends on the direction of driving force as well as GB angles *θ*_1_ and *θ*_2_. *R*(0°, *θ*_2_) and *R*(*θ*_2_, 0°) are plotted in Fig. 4b as a function of *θ*_2_, for nonsymmetric GBs with *θ*_1_ = 0° and **F** parallel to the GB. Similar to the time delay, the reflection is also related to the potential energy barrier at the GB. This is consistent with our observation that reflection preeminently occurs at 5–7 pairs at the GB where the potential barrier is largest (see Fig. 4a).

However, contrary to a transmission process, reflection requires both the stopping and a redirection of an interstitial. Then the reflection rate is the product of the probability *p*_hit_(*θ*_1_, *θ*_2_) for an incoming interstitial to hit a 5–7 pair at the GB and the probability *p*_reflect_(*θ*_1_, *θ*_2_, **F**) for a stopped interstitial to be reflected. *p*_hit_(*θ*_1_, *θ*_2_) is proportional to the density of 5–7 pairs divided by the cosine of the interstitial incidence angle. *p*_reflect_(*θ*_1_, *θ*_2_, **F**) is obtained by solving an equilibrium two-state distribution problem with *p*_reflect_ ∝ exp(*F*_reflect_(*θ*_1_, **F**) *b*/*k*_B_*T*) and *p*_cross_ ∝ exp(*F*_cross_(*θ*_2_, **F**) *b*/*k*_B_*T*). Here *F*_reflect_(*θ*_1_, **F**) and *F*_cross_(*θ*_2_, **F**) are the appropriate projections of **F** to the lattice symmetry directions corresponding to reflection and crossing respectively. Finally, one arrives at the total reflection rate:

2 $${\it{R}}\left( {\theta _1,\theta _2} \right) = {\it{p}}_{{\mathrm{hit}}}\left( {\theta _1,\theta _2} \right){\it{h}}\left( {\left[ {{\it{F}}_{{\mathrm{reflect}}}\left( {\theta _1,{\mathbf{F}}} \right) - {\it{F}}_{{\mathrm{cross}}}\left( {\theta _2,{\mathbf{F}}} \right)} \right]b/{\it{k}}_{\mathrm{B}}{\it{T}}} \right),$$

where *h* is the sigmoid function *h*(*x*) = 1/(*e*^−*x*^ + 1). The projections *F*_reflect_ and *F*_cross_ involve also the normal force exerted by the GB, and are described in the “Methods” section. Given *b* = 4.6 µm, *k*_B_*T* = 4.14 zJ, *θ*_1_ = 0°, and a driving force parallel to the GB with magnitude *F* = 66 fN, the theoretical reflection rates *R*(0°, *θ*_2_) and *R*(*θ*_2_, 0°) as a function of *θ*_2_ are plotted in Fig. 4b alongside the experimental data, showing good agreement. By taking advantage of the large difference of *R*(0°, *θ*_2_) and *R*(*θ*_2_, 0°) at *θ*_2_ > 15°, defects can be strongly localized within a single grain as shown in Fig. 4c and Supplementary Movie 8. The interstitials easily cross the GB from grain 2 to grain 1 but not in the opposite direction, effectively trapping all moving interstitials inside one single region. This is also confirmed by numeric simulations, see Supplementary Movie 9. Note that Fig. 4c shows even direction changes within the grains: this is due to the nonuniform PN barriers and the existence of thermal fluctuations in experiments, and should be distinguished from the reflections that occur at the GBs.

## Discussion

Various techniques have been developed to process materials in order to optimize their properties regarding specific technological applications. Many of these methods, however, are based on empirical findings rather than on a detailed microscopic understanding how defects affect the material properties. This lack of knowledge is partially due to the difficulty to observe the atomistic kinetics of defects moving across GBs with single-particle resolution and in real time. Colloidal monolayers with interstitial defects and externally designed atomic scale GBs, as reported here, provide an ideal two-dimensional emulator where much novel experimental and theoretical understanding can be obtained regarding the GB-defect interactions. While the explored GB topologies are specific to two-dimensional hexagonal crystals, the GBs in three-dimensional metals involve similar patterns of sites with varying coordination and atomic volume which affect the mobility of vacancies and self-interstitials^30–32^. Accordingly, the distortions and splitting of defects by GBs as well as the confinement of defects within certain regions of the polycrystal might also be observed in three-dimensional polycrystals. As GBs and defects are known to assist in annealing of crystal damage^33–36^, the observed channeling of interstitials within polycrystals suggests an intriguing possibility for controlling the damage, failure, and self-healing of materials under mechanical stress. Our experimental approach illustrates the premises for predicting the dynamic behavior under external stress of physical systems characterized by similar topological features, such as the mechanical behavior of two-dimensional materials and nanofriction^37^.

## Methods

### Substrate preparation and characterization

The polycrystalline structure on the sample substrate was created by photolithography. We adopt the algorithm in ref. ^29^ to generate the GB packing geometry. This geometry is transferred to a photo mask, the lattice points on which are opaque circular disks. After exposing the negative photoresist SU8 2000.5 (~500 nm in thickness coated on a glass surface) under the mask, the unexposed disk regions on the photoresist dissolve away in photoresist developers, resulting in circular wells of depths ~500 nm on the photoresist, arranged in a polycrystalline packing. We scanned the SU8 structure under an atomic force microscope with a Bruker OTESPA-R3 tip (tip curvature radius ~7 nm). The scans (Supplementary Fig. 1) show that, for the *b* = 4.60 μm substrate, the diameter of the wells is about 3.6 μm and the depth of the wells is about 550 nm. The values (3.6 μm and 550 nm) can vary slightly (±10%) in experiments from sample to sample.

### Sample preparation

The colloidal suspension is composed of mono-dispersed silica spheres in deionized water. They have a diameter *σ* = 4.28 ± 0.12 μm, buoyant weight *mg* = 348 fN and a gravitational height of *h*_g_ = *k*_B_*T*/*mg* = 11.7 nm at room temperature *T* = 295 K. The colloidal suspension is injected into a sample cell of about 20 mm × 30 mm × 300 μm in size, where 300 μm is the distance from the bottom-patterned substrate to the top cover slide. Under gravity, the particles sediment down to the bottom of the sample cell and uniformly distribute on the substrate which contains regions of flat surface and regions of topographically patterned surface. The colloidal particles are slightly smaller than the lattice spacing of the periodic surface, therefore each well on the periodic surface can host at most one particle. The initial particle coverage (~0.3) is not enough to form a crystalline phase on the periodic surface. To facilitate the formation of crystalline phase on the periodic surface, the sample cell is tilted by 15–20 degrees so that particles can move in from the flat reservoir to the patterned surface. The driving force is such that it is too small to drive isolated particles across the periodic surface, but much larger than needed to drive interstitials across the crystals. Under the driving force, the newly arrived particles from the reservoir will move into the crystalline phase and become interstitials (Supplementary Movie 1). The interstitials will further be driven across the crystalline phase until they reach unoccupied wells. In such a way, the crystalline phase grows larger and larger in the patterned region.

### Particle tracking and interstitial characterization

We recorded experimental images at a 3 Hz frame rate. Using a standard particle-tracking algorithm, we can track the positions of the center of the colloidal particles with a 50 nm accuracy. To calculate the distance *δr* of a particle relative to its equilibrium position (i.e., the nearest well), we fit the positions of all particles in the image to a perfect triangular lattice of lattice spacing *b*, with fitting parameters *x*_0_, *y*_0_, *θ*, where (*x*_0_, *y*_0_) are the coordinates of one of the lattice points and *θ* the lattice orientation. With (*x*_0_, *y*_0_) and *θ*, the position of all other lattice points are calculated. *δr* of a particle is then the distance between the particle and the nearest lattice point. An interstitial is defined as a cluster of close-packed particles whose *δr* > 1.2 μm. The position of an interstitial is the center-of-mass position of the cluster. The velocity of individual interstitials can then be calculated from the time series of their center-of-mass displacements.

### Calculation of average delay time

To calculate the average delay time of interstitials crossing symmetric GBs with angle *θ*, as shown in Fig. 3b, we measure the average velocity *v*(*θ*) of interstitials crossing the GB and traveling for an horizontal distance Δ*x*. As a reference, we take the average velocity for interstitials in a single crystal—i.e., *v*(*θ* = 0). The average delay time is then defined to be: Δ*x*/*v*(*θ*) − Δ*x*/*v*(0). Δ*x* = 300 μm in both experiments and simulations. To calculate *v*(*θ*) in experiments, the average velocity *v*_p_(*θ*) of each colloidal particle in the field of view during a time period of ~1000 s is measured. Then, *v*(*θ*) = *N*_p_*v*_p_(*θ*)/*N*_int_, where *N*_p_ ~ 3500 is the number of particles and *N*_int_ = Σ_i_*w*_i_ ~ 100 is the total number of interstitials (weighted by their width *w*_i_). *w*_i_ ≥ 3 in most cases. The calculated *v*(*θ*)/*v*(0) is shown in Supplementary Fig. 5 for both experiments and simulations. *v*(*θ*)/*v*(0) reaches a minimum around *θ* = 15°, corresponding to the longest delay of interstitials.

### Determination of pile-up ratio of interstitials

We define the pile-up ratio as *p* = (∫_−Δ*x*/2<*x*<0_*δr*^2^(*x*)d*x* − ∫_0<*x*<Δ*x*/2_*δr*^2^(*x*)d*x*)/∫_−Δ*x*/2<*x*<Δ*x*/2_*δr*^2^(*x*)d*x*, i.e. the difference of the shaded areas at *x* < 0 and at *x* > 0 in Supplementary Fig. 6, normalized by the entire shaded area, where *δr*^2^(*x*) = 1/*Y* × ∫_0<*y*<Δ*y*_ *δr*^2^(*x*,*y*)d*y* is the *y*-averaged *δr*^2^, *Δx* = 300 μm and *Δy* = 240 μm.

### Evaluation of the grain boundary defect density

The precise local structure of the GBs depends on the alignment of the two neighboring grains relative to the direction of the GB itself. We can predict very accurately the average defect concentration and its composition in terms of topological defects. As shown in Supplementary Fig. 7, due to the lattice misalignment, the number of lattice lines joining at the GB from the left is generally different from the number of lattice lines joining from the right. To account for this discrepancy, one 5–7 pair is generated at the GB for each lattice line difference. Using this simple geometric observation, we propose simple formulas to describe the density of 5–7 pairs in any GB between triangular lattices. In general, there are only two independent ways (highlighted in red and blue in Supplementary Fig. 7) for the lattice lines in the two grains to join at the GB with minimum deflection, therefore there are two sets of 5–7 pairs at the GB. For one set, the (average) distance between 5–7 pairs is *D*′ = √3/2 *b*/(cos(*θ*_1_) − cos(*β*)); for the other set, the distance between 5–7 pairs is *D*″ = √3/2 *b*/(cos(*θ*_1_ + 60°) − cos(*β* − 60°)). The angle *β* depends on the relative orientation of the two grains and is related to which directions present minimal deflection: *β* = *θ*_2_ if *θ*_1_ + *θ*_2_ < 30° and *β* = 60° − *θ*_2_ if *θ*_1_ + *θ*_2_ > 30°. The total density of 5–7 pairs is then *ρ*_p_ = 1/*D*′ + 1/*D*″. We find that these formulas accurately describe the structural properties of the GBs constructed according to the algorithm of ref. ^29^. The value of *ρ*_p_ is 0 at *θ*_1_ + *θ*_2_ = 0° and 60° and reaches the maximum value near *θ*_1_ + *θ*_2_ = 30°. More illustrations of GBs and their 5–7 pairs with different values of *θ*_1_ and *θ*_2_ are shown in Supplementary Fig. 8.

### Reflection and transmission analysis

In a first approximation a GB can be considered as a barrier that exerts a normal force onto approaching interstitials. See Supplementary Fig. 9, a driving force **F** parallel to the GB, and with magnitude *F*, has component *F*_crossing_ = *F* cos(30° + *θ*_2_) in the direction of transmission, and *F* cos(30° + *θ*_1_) in the direction of reflection. The interstitial is attracted to the GB in the direction of the incoming lattice line with a force equal to *P* = *F* cos(30° − *θ*_1_). The resulting normal force, approximating the GB as a hard wall, is *N* = *F* cos(30° − *θ*_1_) sin(30° − *θ*_1_). When this normal force is projected back to the reflection direction, it gives a contribution *F* cos(30° − *θ*_1_) sin(30° − *θ*_1_) cos(60° − *θ*_1_). Consequently, the total force in the direction of reflection is *F*_reflect_ = *F* cos(30° + *θ*_1_) + *F* cos(30° − *θ*_1_) sin(30° − *θ*_1_) cos(60° − *θ*_1_). This quantity is an ingredient of Eq. (2).

### Modeling and molecular dynamics

The substrate corrugation *V*(**r**) explored by a single particle at position **r** is the sum of terms *V*_dimple_(|**r** − **r**_**i**_|), where **r**_i_ are the centers of the individual potential wells, as placed in a regular two-dimensional lattice or, near a GB, according to the Voronoi algorithm^29^. *V*_dimple_(|**r**|) is a smooth approximation of the potential-energy profile for a sphere of radius *R* located at a distance **r** from the center of a cylindrical well. To avoid cusps in the potential energy, we use *V*_dimple_(**r**) = −*ϵ* for |**r**| < *r*_m_; *V*_dimple_(**r**) = −*ϵ*/2 tanh((*ξ* − *w*_d_)/(*ξ*(1 − *ξ*)) − 1.0) for *r*_m_ < |**r**| < *r*_M_; *V*_dimple_(**r**) = 0 for |**r**| > *r*_M_. Here, *ξ* = (*r* − *r*_m_)/(*r*_M_ − *r*_m_). The parameters *w*_d_ = 0.29*, r*_m_ = 0.6 µm and *r*_M_ = 2.0 µm have been fitted to best replicate the experimental profile experienced by the *σ* = 4.28 μm spheres on the *b* = 4.6 µm substrate. We adopt an energy corrugation depth *ϵ* = 170 zJ, consistent with a well depth of ~500 nm. For the investigation of the colloidal-particles dynamics, we use a sum of two-body interaction potentials of the form *v*_int_(|**r**_i_ − **r**_j_|) = +∞ for |**r**_i_ − **r**_j_| < *r*_0_; *v*_int_(|**r**_i_ − **r**_j_|) = *v*_LJ_(|**r**_i_ − **r**_j_| − *r*_0_) for |**r**_i_ − **r**_j_| > *r*_0_. This combines a hard-core repulsion at distances smaller than *r*_0_ = 1.0 µm and a softer Lennard-Jones repulsion at larger distances, with *ϵ*_LJ_ = 1 zJ, *σ*_LJ_ = 3.6 µm and a “truncated and shifted force” cutoff at 1.6 *σ*_LJ_. The parameters have been chosen to fit structural properties of experimental interstitials. We perform *T* = 0 Langevin dynamics with a damping rate *γ* = 20.0 ms^−1^, within a fourth-order Runge–Kutta integration scheme.

## Supplementary information

Supplementary Information

Description of Additional Supplementary Files

Supplementary Movie 1

Supplementary Movie 2

Supplementary Movie 3

Supplementary Movie 4

Supplementary Movie 5

Supplementary Movie 6

Supplementary Movie 7

Supplementary Movie 8

Supplementary Movie 9

## Data Availability

The data that support the findings of this study are available from the corresponding author upon request.
