# Supplementary material for: Pile-up transmission and reflection of topological defects at grain boundaries in colloidal crystals
Source: Nat Commun. 2020 Jun 17;11:3079. doi: 10.1038/s41467-020-16870-w (PMC7300131; doi:10.1038/s41467-020-16870-w)
Supplement: Supplementary file 1 — Supplementary Information [file 41467_2020_16870_MOESM1_ESM.pdf]

Supplementary Information
for
Pile-up transmission and reflection of topological defects at grain boundaries in colloidal crystals

Xin Cao[1], Emanuele Panizon[1], Andrea Vanossi[2,3], Nicola Manini[4], Erio Tosatti[2,3,5], Clemens Bechinger[1]*

1. Fachbereich Physik, Universität Konstanz, 78464 Konstanz, Germany
2. International School for Advanced Studies (SISSA), Via Bonomea 265, 34136 Trieste, Italy
3. CNR-IOM Democritos National Simulation Center, Via Bonomea 265, 34136 Trieste, Italy
4. Dipartimento di Fisica, Università degli Studi di Milano, Via Celoria 16, 20133 Milano, Italy
5. The Abdus Salam International Centre for Theoretical Physics (ICTP), Trieste, Italy

*Corresponding author: clemens.bechinger@uni-konstanz.de

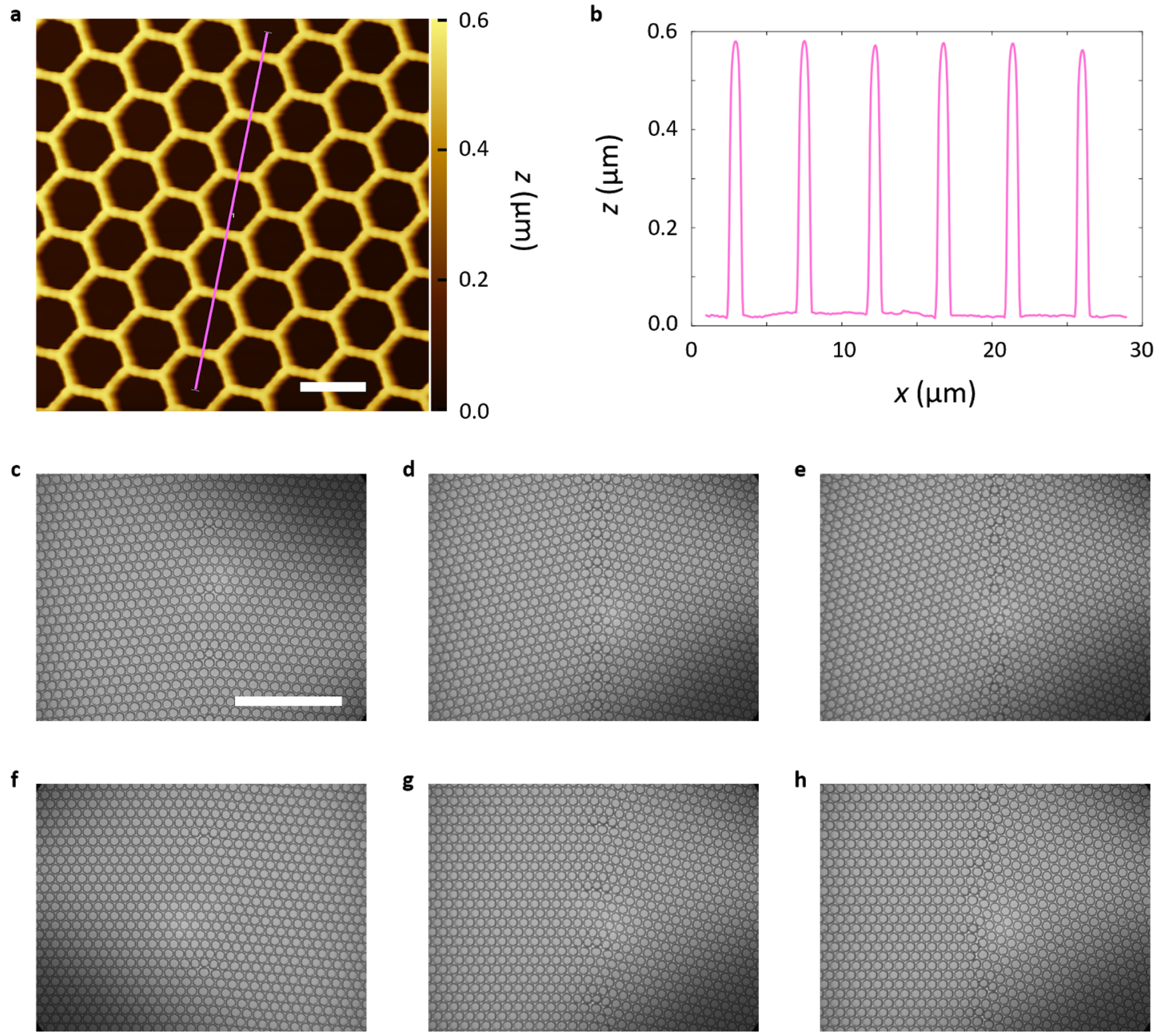

Supplementary Figure 1. Characterization of periodic surfaces and grain boundaries. (**a**) Atomic force microscopy scan of a lithographically patterned substrate with b = 4.60 µm. Scale bar is 5 µm. (**b**) Height profile along the pink line marked in (**a**). (**c,d,e**) Optical-microscope images showing patterned surfaces with symmetric GBs characterized by $\Theta_1 = \Theta_2$ = 4.72°, 10.89° and 19.11°, respectively. (**f,g,h**) Patterned surfaces with GBs characterized by Θ_1 = 0° and Θ_2 = 4.72°, 10.89° and 19.11°, respectively. Scale bar is 50 µm.

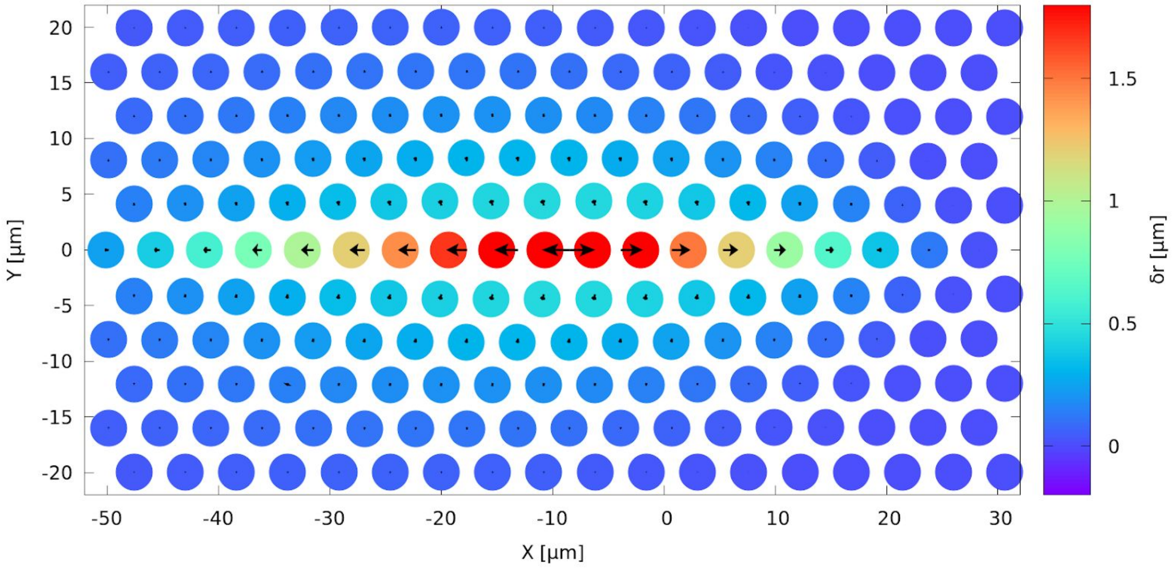

Supplementary Figure 2. Evidence of lateral friction. Displacements of particle positions from their corresponding equilibrium positions near a moving interstitial obtained from simulations. The motion of an interstitial can noticeably displace particles which are 3 lines away on both sides even though the particle-particle interaction is short ranged. The distortion leads to a lateral friction which slows down the motion of interstitials.

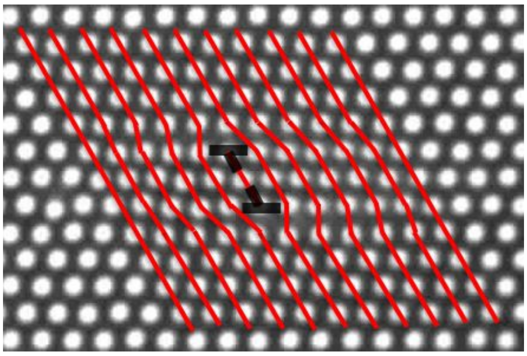

Supplementary Figure 3. Crystallographic nature of the interstitial defects. The crystallographic nature of the interstitial defect of Fig. 1**b**. Red lines indicate lattice directions. The associated pair of dislocations (labeled with thick black '⊥') with opposite Burgers vectors is highlighted.

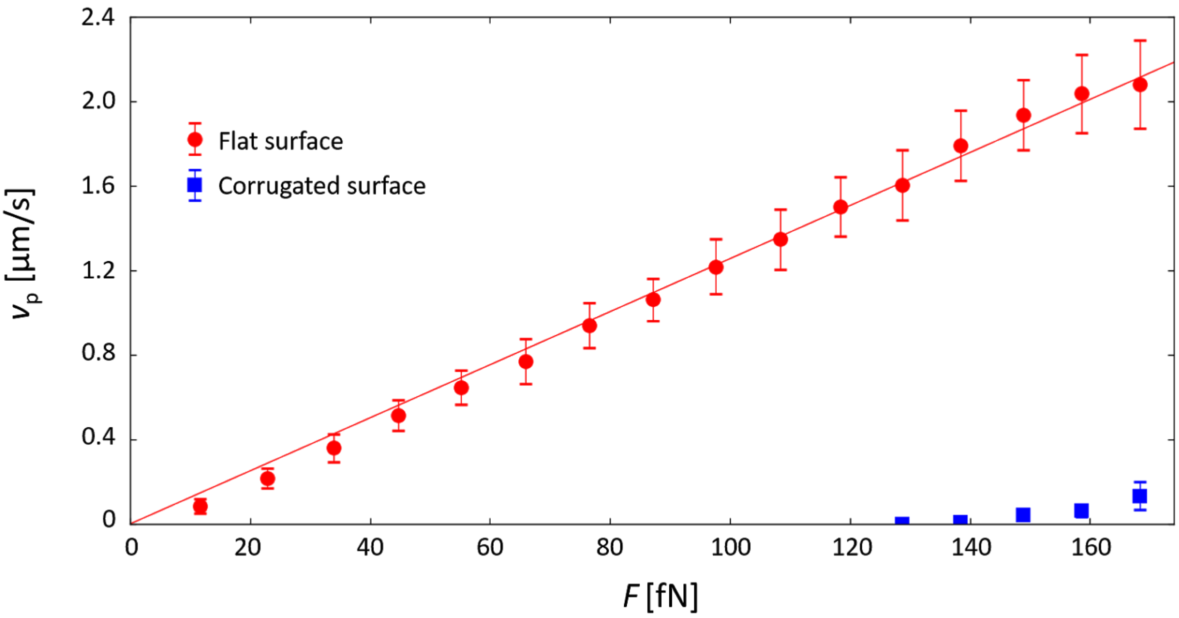

Supplementary Figure 4. Velocity of single particles. Velocity of single isolate particles on the flat surface (circles) as well as on the corrugated surface (squares, b = 4.6 µm) as a function of the driving force F. On the flat surface, the particle velocity follows the relation $v_p = \mu F$, where μ = 0.0126 $\mu m \cdot s^{-1} \cdot fN^{-1}$ is the fitted mobility (solid line). In contrast, on corrugated surfaces, the particle remains pinned until F reaches a critical value ~150 fN. This critical force is much larger than the critical force of interstitials (10~20 fN) on the same surface. The maximum force experimentally accessible in the setup is ~170 fN. Error bars are standard deviations of the data points.

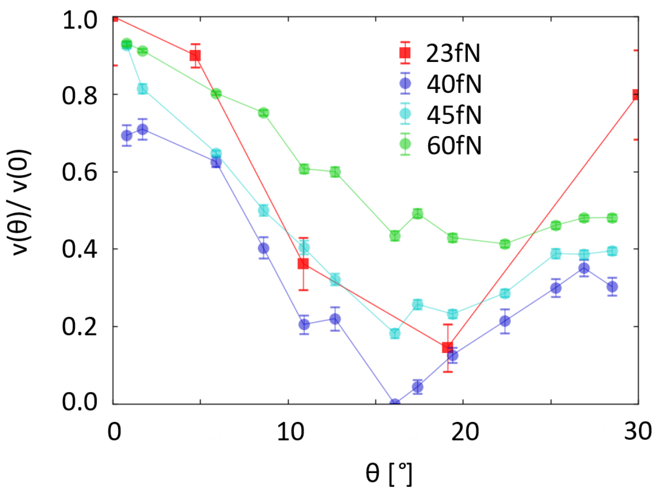

Supplementary Figure 5. Slowing down of interstitials by symmetric GBs. Normalised interstitial average velocity $v(\Theta)$ / $v(0)$ as a function of GB angle θ in experiments and in simulation at different driving force F, given that the interstitials have to cross a GB and travel a total horizontal distance Δx = 300 µm. Squares are experimental data and circles are simulation data. Error bars are standard deviations of the data points.

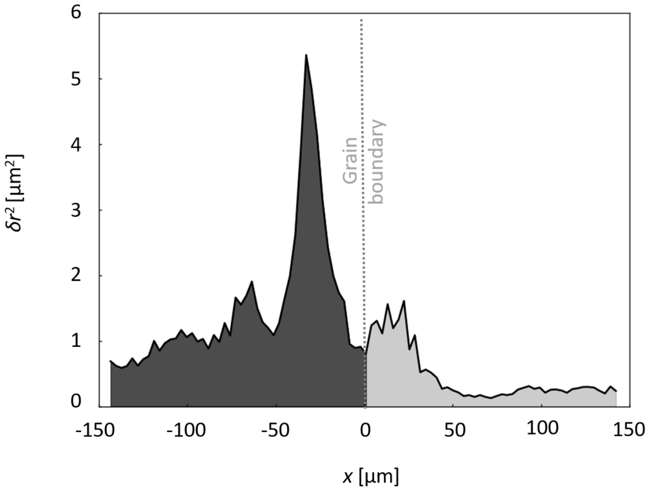

Supplementary Figure 6. Pile-up ratio. y-averaged experimentally observed mean square displacements from equilibrium positions δr^2 as a function of horizontal axis x position when interstitials start to pile up against a GB placed at x = 0 with Θ = 19.1°, under F = 23 fN. The pile up ratio is defined as p = $(\int_{-\Delta x/2<x<0}\delta r^2(x)\mathrm{d}x$ - $\int_{0<x<\Delta x/2}\delta r^2(x)\mathrm{d}x)$ / $\int_{-\Delta x/2<x<\Delta x/2}\delta r^2(x)\mathrm{d}x$, i.e. the normalised difference of the shaded areas at x < 0 and at x > 0.

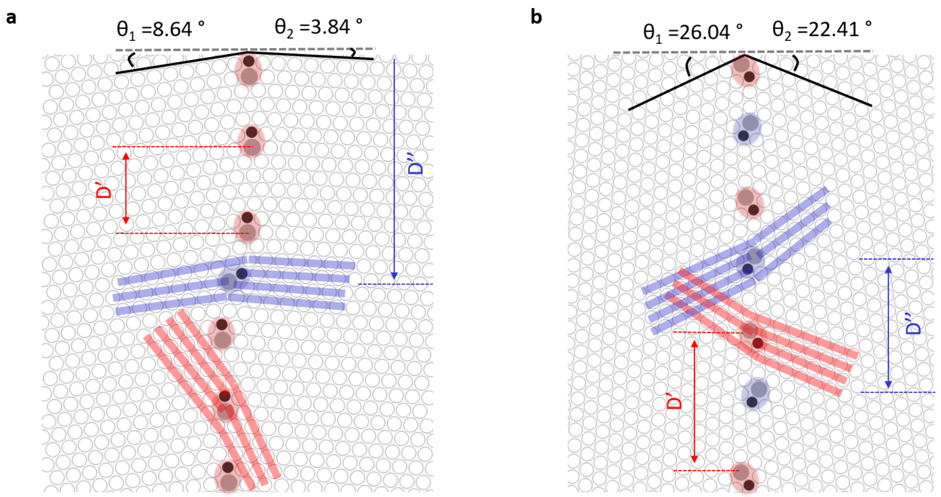

Supplementary Figure 7. Two sets of 5-7 pairs at GBs. Illustrations of GBs with (**a**) $\theta_1+\theta_2<30°$ and (**b**) $\theta_1+\theta_2>30°$. Blue and red lines correspond to the two independent ways for the lattice lines in the two grains to meet at the GB. The different colors correspond to defects with average distances *D*' and *D*'' respectively. *D*'' is much larger than *D*' in (**a**) due to the small values of θ_1 and θ_2, therefore only one blue 5-7 pair is shown in (**a**).

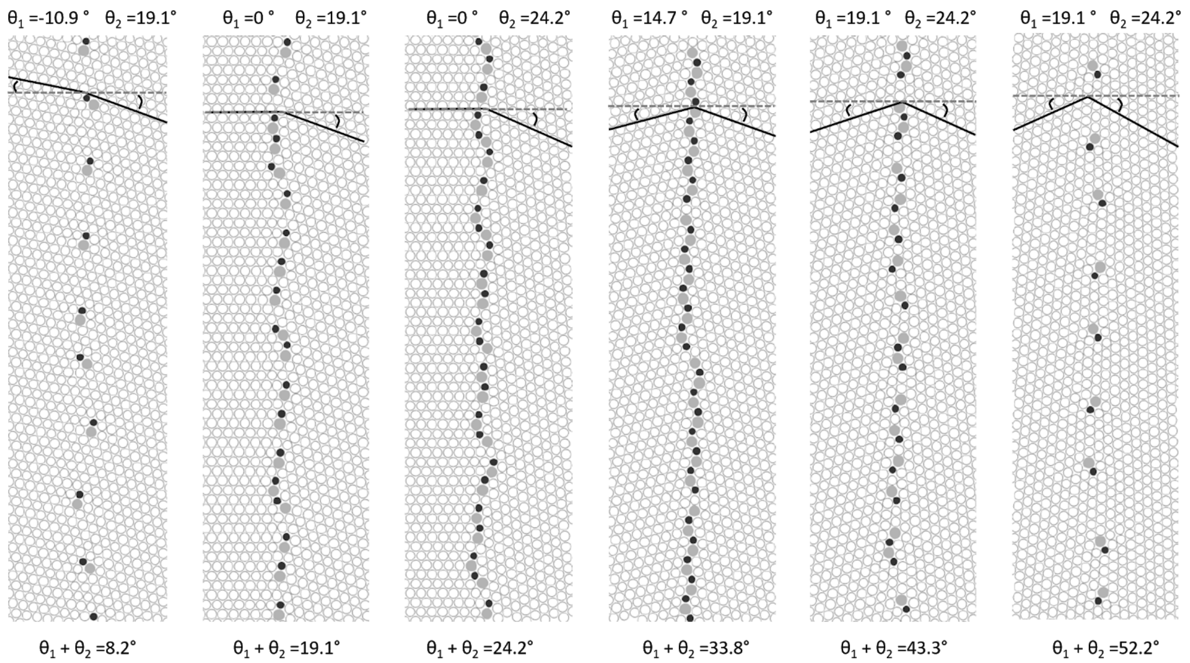

Supplementary Figure 8. Grain boundary defects. Illustration of pairs of pentagonal (dark) and heptagonal (gray) defects at GBs, characterized by various relative orientation angles θ_1 and θ_2, as indicated.

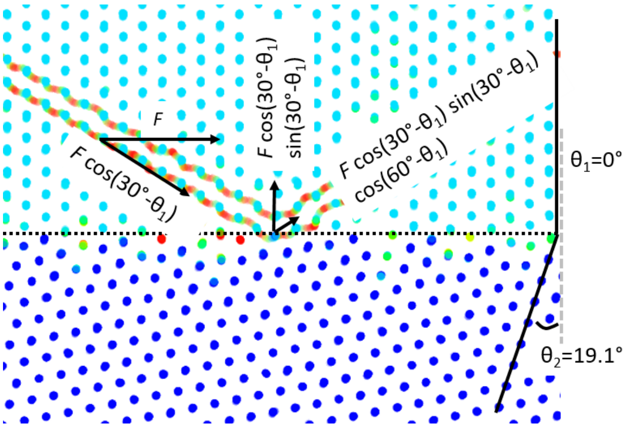

Supplementary Figure 9. Projection of forces for interstitial reflections at GBs. Illustration of the decomposition of the driving force in the direction of the incoming interstitial, the resulting normal force exerted on the interstitial by the GB and its contribution to $F_{reflect}$, for a GB with $\Theta_1 = 0°$ and $\Theta_2 = 19.1°$.
