# Supplementary material for: Pile-up transmission and reflection of topological defects at grain boundaries in colloidal crystals
Source: Nat Commun. 2020 Jun 17;11:3079. doi: 10.1038/s41467-020-16870-w (PMC7300131; doi:10.1038/s41467-020-16870-w)
Supplement: Supplementary file 2 — Description of Additional Supplementary Files [file 41467_2020_16870_MOESM2_ESM.docx]

Description of Additional Supplementary Files

**Title:** Supplementary Movie 1.

**Description:** Colloidal particles (σ = 4.28 μm) are injected into a crystal phase (b = 4.6 μm) and form interstitials which propagate rapidly along lattice directions. F = 87 fN in the horizontal direction from left to right. Movie acceleration: 8 × real time.

**Title:** Supplementary Movie 2.

**Description:** Two interstitials moving in different lattice directions start to mutually align when they approach each other. In experiment F = 55 fN horizontally from left to right. Movie acceleration: 8 × real time.

**Title:** Supplementary Movie 3.

**Description:** A simulation showing several collisions between interstitial defects. After collision, the interstitial defects aggregate and their overall velocity increases.

**Title:** Supplementary Movie 4.

**Description:** A moving w = 2 interstitial pushes a co-linear static w=1 interstitial forward. F = 23 fN left to right. Movie acceleration: 8 × real time.

**Title:** Supplementary Movie 5.

**Description:** A w = 1 interstitial moving through the θ1 = θ 2 = 4.72° GB along line 4 (without hitting 5-7 pairs) and line 1 (hitting at a 5-7 pair) respectively. F = 45 fN left to right. Movie acceleration: 8 × real time.

**Title:** Supplementary Movie 6.

**Description:** Distortion and splitting of a w = 14 interstitial moving through the θ 1 = θ 2 = 4.72° GB. F = 45 fN left to right. Movie acceleration: 8 × real time.

**Title:** Supplementary Movie 7.

**Description:** Pile up of interstitials in front of a θ 1 = θ 2 = 19.1° GB. F = 23 fN left to right. Movie acceleration: 8 × real time.

**Title:** Supplementary Movie 8.

**Description:** Confinement of interstitials in stripe-shaped polycrystals. The interstitials are trapped in the middle stripe. The four GBs from bottom to top have GB angles (0°,-19.1°), (19.1°,0°), (0°,19.1°) and (-19.1°,0°) respectively. F = 66 fN left to right. Movie acceleration: 24 × real time.

**Title:** Supplementary Movie 9.

**Description:** A simulation of the confinement of interstitials in stripe-shaped polycrystals. The interstitials tend to become trapped inside the middle stripe. The four GBs from bottom to top have GB angles (0°,-17.6°), (17.6°,0°), (0°,17.6°) and (-17.6°,0°) respectively. The starting configuration has 121 extra particles, of which 120 initially immobile and situated at the 7-coordinated defects of the GBs. At t = 0 s, one interstitial is situated far from the GBs, mobile under a driving force of F = 95 fN. As the simulation progresses, the mobile interstitial detaches other interstitials from the GBs, which in turn become mobile and trigger more interstitials. The system is simulated under periodic boundary conditions in both x and y directions (size 501 μm × 456 μm).
